# Saphenous Vein Graft Pseudoaneurysm Repair with GraftMaster®

**DOI:** 10.7759/cureus.3591

**Published:** 2018-11-14

**Authors:** Qasim Jehangir, Charles Lambert, Asad Sawar

**Affiliations:** 1 Cardiology, Hospital of the University of Pennsylvania, Philadelphia, USA; 2 Cardiology, Florida Hospital Tampa Pepin Heart Institute, Tampa, USA

**Keywords:** pseudo-aneurysm, coronary artery bypass surgery, per cutaneous coronary intervention, covered stents

## Abstract

Saphenous vein graft (SVG) pseudoaneurysm is a rare complication of coronary artery bypass graft (CABG) surgery. Despite the high mortality associated with SVG pseudoaneurysm, there is no consensus on the optimal management of these pseudoaneurysms as they are infrequently reported in the literature. We report a case of a 55-year-old man with prior CABG surgery who presented with cough associated with hemoptysis and chest pain, and was found to have SVG pseudoaneurysm. The pseudoaneurysm was successfully closed with polytetrafluoroethylene (PTFE)-covered Jostent GraftMaster^®^ (Abbott Vascular, Santa Clara, CA). We propose that GraftMaster is an effective means of treating SVG pseudoaneurysms percutaneously.

## Introduction

Saphenous vein graft (SVG) pseudoaneurysm is a rare postoperative complication of coronary artery bypass graft (CABG) surgery with an incidence estimated to be <1% and mortality as high as 12.5% [1-2]. The presentation can vary from an incidental finding on imaging to symptoms of angina, heart failure, hemoptysis, rupture, and sudden death in some cases. There are no clear guidelines on the optimal management of SVG pseudoaneurysms as they are infrequently reported in the literature. Herein, we present a case of SVG pseudoaneurysm that presented six months after CABG and was successfully treated with Jostent GraftMaster® (Abbott Vascular, Santa Clara, CA).

## Case presentation

**Figure 1:**
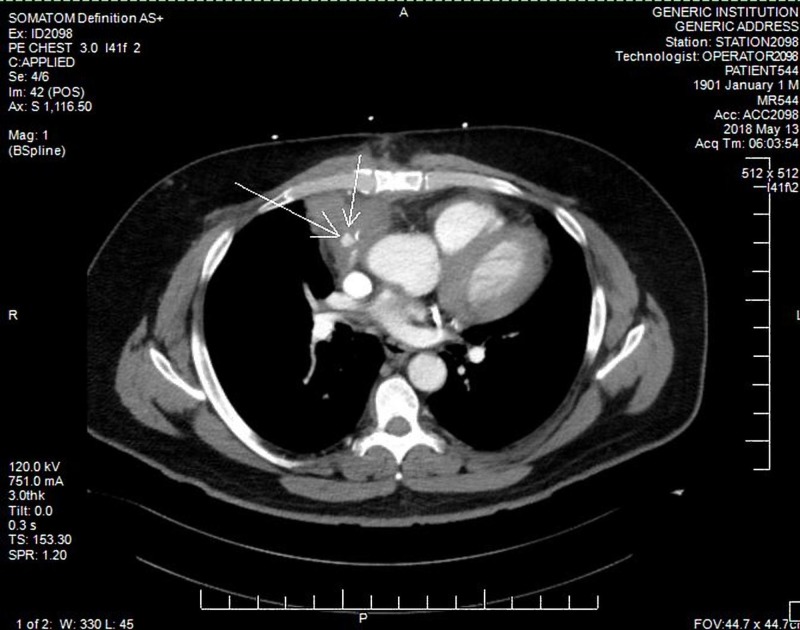
Computed tomography scan of the chest with contrast showing pseudoaneurysm in saphenous vein graft to right coronary artery, 2 cm from its origin, measuring 1.2 cm in size with adjacent fluid, possibly representing hemorrhagic debris.

A 55-year-old man with a history of hypertension, hyperlipidemia, coronary artery disease, status post CABG six months ago with SVGs to the obtuse marginal and right coronary arteries (RCAs), and left internal mammary artery graft to the left anterior descending coronary artery, presented to the emergency department with two episodes of cough with hemoptysis associated with some chest discomfort. Both episodes resolved spontaneously. The patient was hemodynamically stable. Laboratory evaluation included hemoglobin of 12.2 g/dL and normal troponin. Electrocardiogram did not show any changes suggestive of cardiac ischemia. A computed tomography (CT) scan of the chest with contrast to rule out pulmonary embolism showed pseudoaneurysm in SVG graft to RCA, 2 cm from its origin, measuring 1.2 cm in size with adjacent fluid possibly representing hemorrhagic debris (Figure 1). The patient was hospitalized for further management.

**Figure 2:**
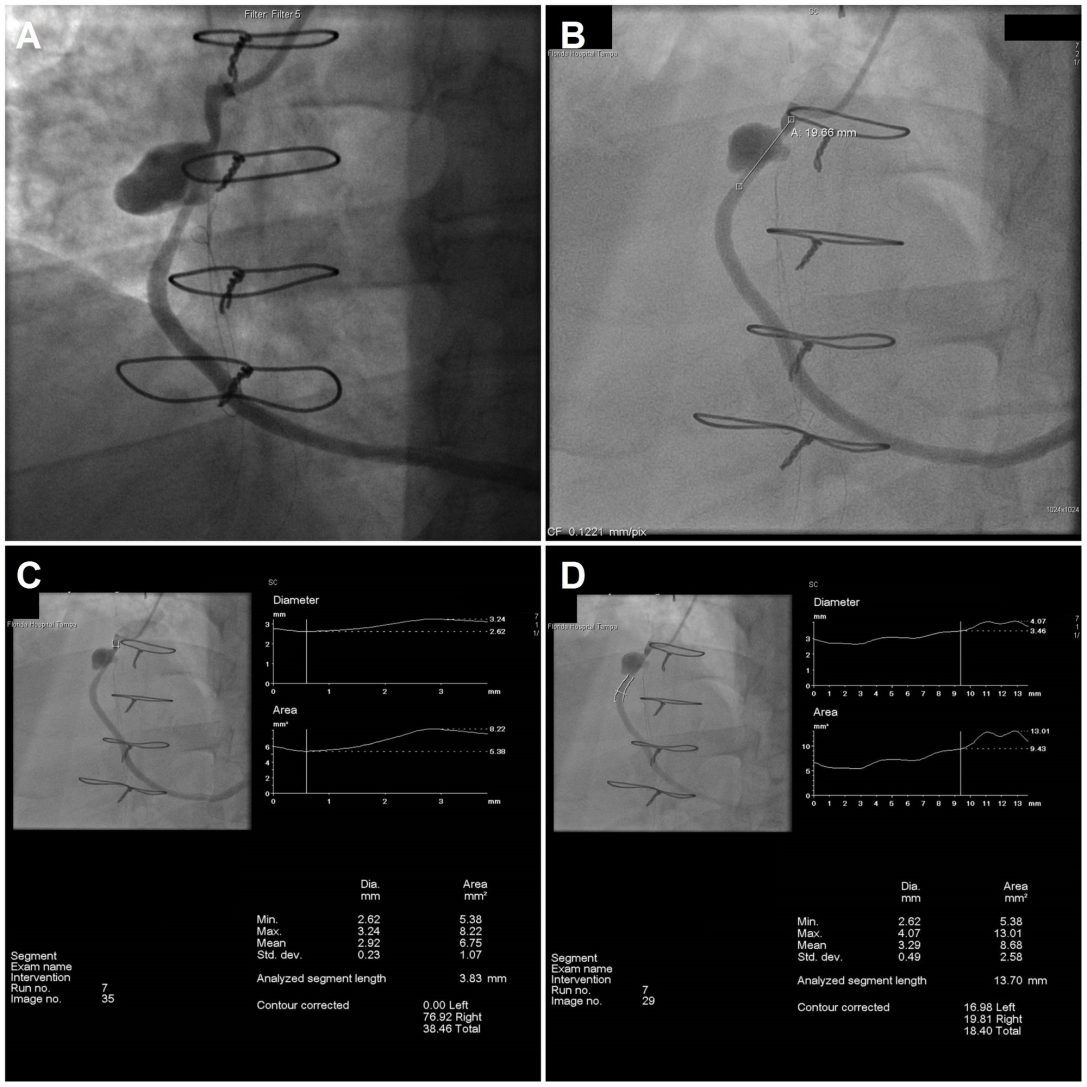
A-D. Angiogram showing pseudoaneurysm in saphenous vein graft to right coronary artery measuring 2 cm in size and 70% stenosis in the proximal third of the graft.

**Figure 3:**
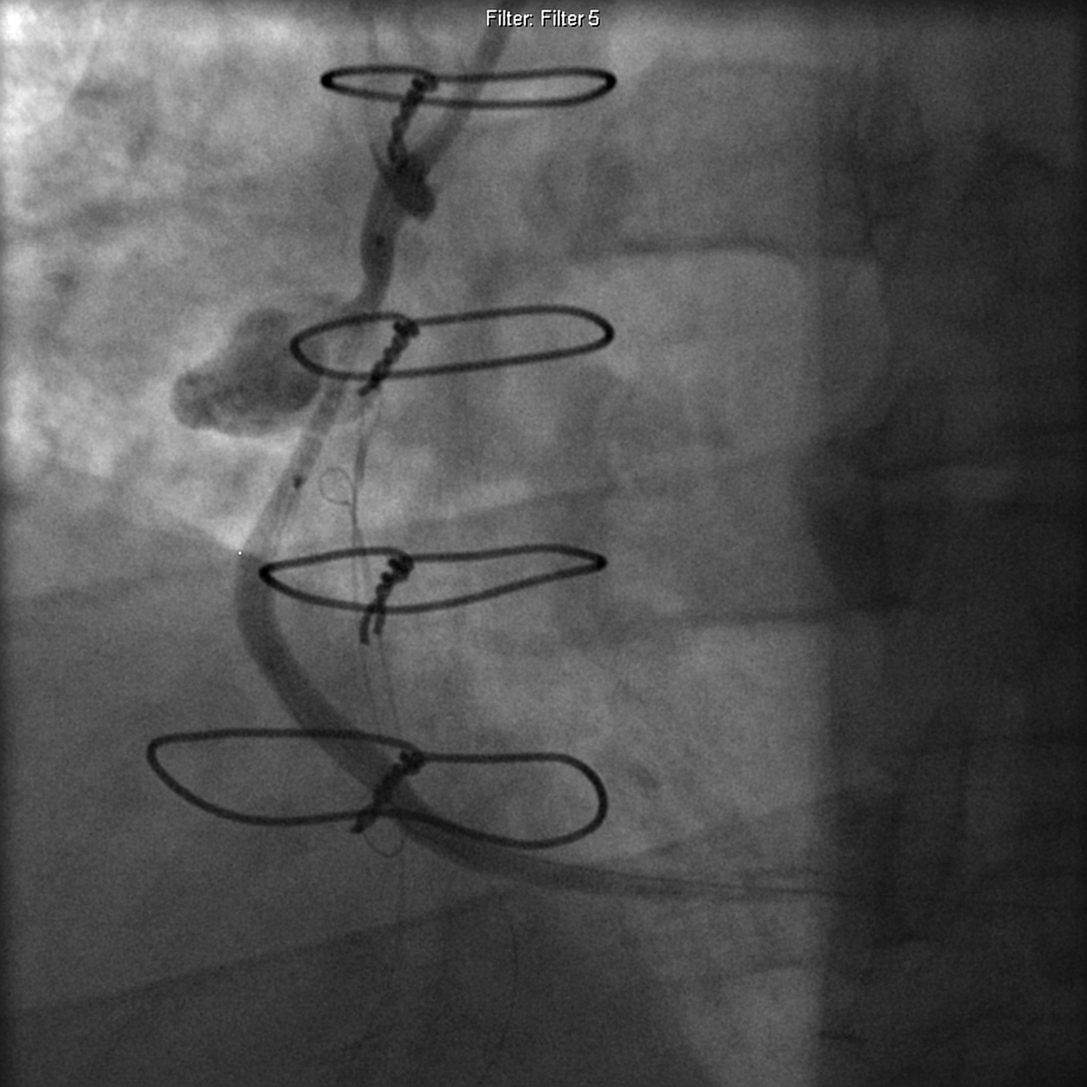
Balloon angioplasty using NC Emerge 4.0 mm × 15 mm balloon in saphenous vein graft to right coronary artery.

**Figure 4:**
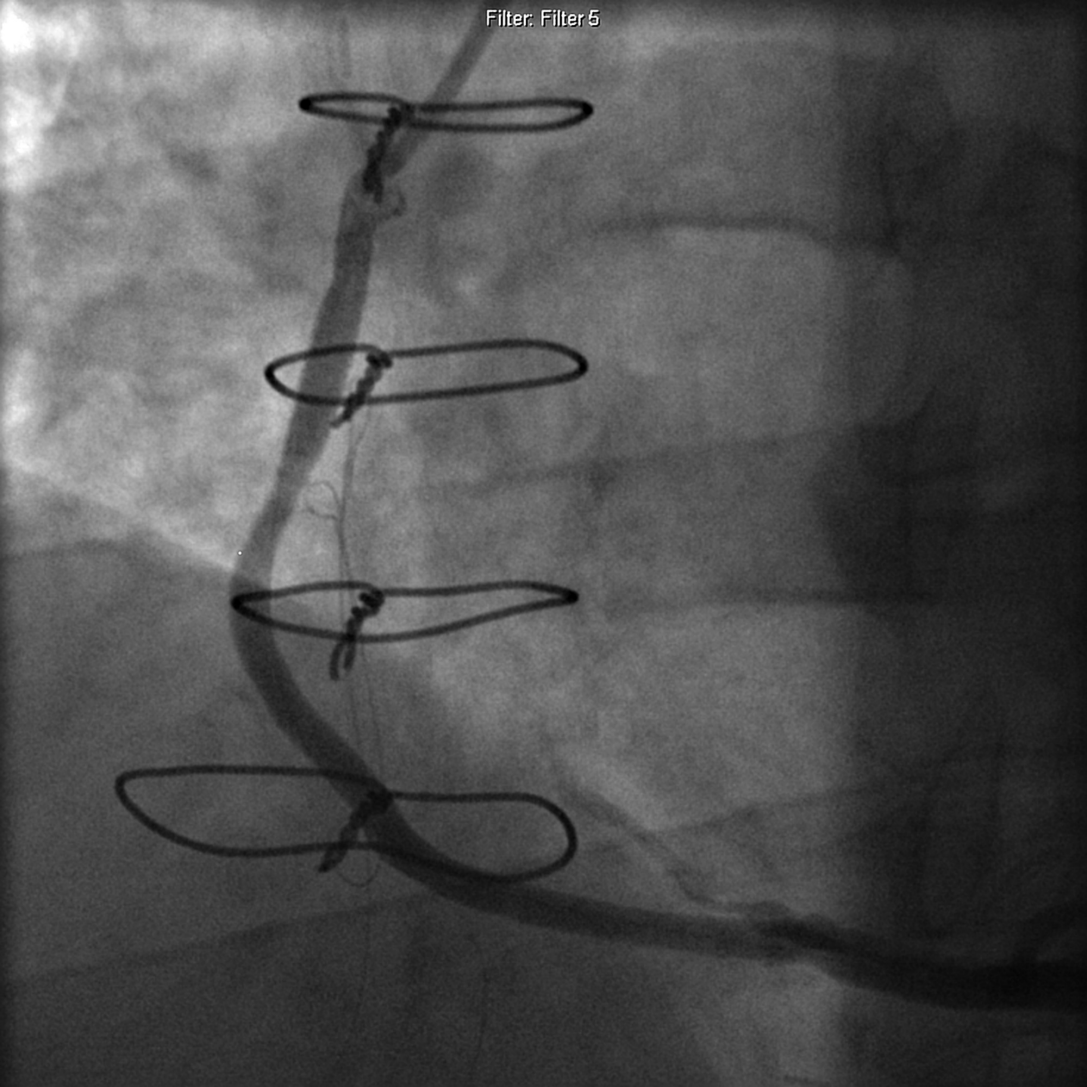
Angiogram showing 4.0 mm × 26 mm GraftMaster® covering 99% of the lesion in the saphenous vein graft with 0% residual stenosis.

The decision was made to repair the pseudoaneurysm through percutaneous approach with polytetrafluoroethylene (PTFE)-covered Jostent GraftMaster after a multi-disciplinary meeting. Appropriate permission was obtained for GraftMaster use. The patient was brought to the catheterization laboratory and left femoral access was obtained using modified Seldinger technique. FR4 7 Fr guiding catheter was advanced to aorta and positioned at the aortic anastomosis of the graft under fluoroscopic guidance. Angiography was performed in multiple locations using hand-injection of contrast. The SVG graft to RCA revealed pseudoaneurysm measuring 2 cm in size and 70% stenosis in the proximal third of the graft (Figure 2, Video 1). A BMW 0.014” 190CM J-Tip wire was used to cross the lesion. Balloon angioplasty was performed using NC Emerge 4.0 mm × 15 mm balloon with single inflation and a maximum inflation pressure of 15 atm (Figure 3, Video 2). Intracoronary stenting was performed with 4.0 mm × 26 mm GraftMaster and deployed at a maximum inflation pressure of 55 atm. Stent covered 99% of the lesion in the SVG and there was 0% residual stenosis (Figure 4, Video 3). There was thrombolysis in myocardial infarction (TIMI) three flow before and after the procedure. There were no procedural complications, and the patient was continued on dual antiplatelet therapy. One day postoperatively, the patient was discharged home in stable condition, with a follow-up angiogram scheduled in six months. The patient continued to do well without any new complaints.

**Video 1:** Angiogram showing pseudoaneurysm in saphenous vein graft to right coronary artery.

**Video 2:** Balloon angioplasty using NC Emerge 4.0 mm × 15 mm balloon in saphenous vein graft to right coronary artery.

**Video 3:** Angiogram showing 4.0 mm × 26 mm GraftMaster® covering 99% of the lesion in the saphenous vein graft with 0% residual stenosis.

## Discussion

Aneurysmal dilation of SVG following CABG is an infrequent occurrence that can either be a true aneurysm (SVGA) or a pseudoaneurysm with pseudoaneurysms being six times more common than SVGAs [3]. The wall of SVGA contains all three vascular layers (intima, media, and adventitia), whereas the wall of pseudoaneurysm is devoid of at least one of these layers. The site of aneurysmal dilation can provide information about the diagnosis. SVGA commonly involves the body of the graft, whereas, SVG pseudoaneurysms usually occur at the anastomotic site. SVGAs are typically fusiform and thought to be atherosclerotic in nature. The initial likely event is atheroma formation leading to plaque rupture and injury to the vessel wall which is exacerbated by arterial pressures within the vein graft. Hypertension and hyperlipidemia can accelerate arteriosclerosis and the formation of an SVGA. The vicinity of venous valves and branching sites is especially prone to SVGA formation. Another possible contributing factor can be the damage during the initial surgical procedure [4]. The SVG pseudoaneurysms are saccular and thought to occur secondary to imperfect suture placement, tension on the anastomosis resulting in suture rupture, or trauma to the graft like in our case where the proposed cause was trauma secondary to temporary epicardial pacer wire. Infections and chronic corticosteroid use can also be associated with SVG pseudoaneurysm formation. The RCA graft is the most commonly affected in pseudoaneurysms (47%), followed by left circumflex artery (29%) and left anterior descending artery (20%) [2]. Many patients with SVG pseudoaneurysms are asymptomatic and incidentally detected on chest imaging; others can present with angina, myocardial infarction, heart failure, mass effect, fistula formation, rupture, hemoptysis, hemothorax, and/or sudden death. The average time to presentation is several years, however, SVG pseudoaneurysms can present as early as a few days. The aneurysms usually increase in size with time, although the rates of growth are variable. The rates of adverse events such as fistula formation, compression of adjacent structures, myocardial infarction, rupture, and death typically increase with conservative management as the size of the aneurysm increases. The risk of these adverse events is 33.3% for aneurysms measuring 20 mm, increasing to as high as 69.2% for aneurysms >100 mm [5]. Because of the potential lethality of the condition and variability in presentation, a high index of suspicion is required for early diagnosis. As chest X-ray and echocardiogram findings can be nonspecific, advanced imaging with CT and cardiac magnetic resonance imaging is required for correct diagnosis, ruling out complications and guiding further therapy [5]. In our case, CT scan showed pseudoaneurysm in SVG to RCA which was consistent with the findings of coronary angiogram.

The optimal approach to treating patients with SVGA pseudoaneurysms is not well defined. The goals of therapy are to reduce the risk of complications and improve long-term survival. Treatment options include medical management with close surveillance, surgical repair, and percutaneous closure with covered stents, coil embolization, and Amplatzer vascular plug in the neck of aneurysm [4, 6-8]. No randomized controlled or prospective trials have been conducted to compare the efficacy of one treatment option with another. Medical management with close observation can be an option in patients who are asymptomatic and have no evidence of myocardial ischemia. Antianginal therapy with beta blockers and nitrates may provide relief in patients with ischemic symptoms. Antiplatelet and antithrombotic therapies can prevent possible complications of thrombosis and distal embolization. However, no standard medical therapy is recommended for these pseudoaneurysms [9]. Most patients require early percutaneous or surgical repair to prevent expansion with possible rupture and mortality. Endovascular approach is a safe option in high-risk patients who are poor surgical candidates. Coil embolization and vascular plug insertion are often considered when the affected graft is occluded. The SVG in our patient was patent, so a covered stent was considered a suitable option to help maintain the patency of distal flow. Moreover, our patient was only six months out of CABG and cardiothoracic surgery did not recommend reintervention, so it was decided to close the pseudoaneurysm percutaneously through a covered stent.

GraftMaster Coronary Stent Graft System is approved under Humanitarian Device Exemption for the repair of free perforations in native coronary vessels and SVG. There are some reported cases of repair of native coronary artery aneurysms and perforations with GraftMaster [10-11]. However, it has been rarely used for the repair of SVG pseudoaneurysms [12]. Here we report a case of 2 cm SVG pseudoaneurysm that presented six months after the CABG surgery. The patient was successfully treated with GraftMaster. The pseudoaneurysm closure was complete, and the patient had a good clinical outcome postoperatively.

## Conclusions

Pseudoaneurysm formation in SVG is an infrequent but life-threatening complication of CABG surgery which requires a high index of suspicion for diagnosis. Early detection and treatment of these pseudoaneurysms can reduce morbidity and mortality. GraftMaster is an effective means of treating SVG pseudoaneurysms percutaneously. Future comparative studies on the long-term safety and efficacy of GraftMaster along with other treatment options for SVG pseudoaneurysms will be useful.
